# Dr. James W. Casey

**Published:** 1908-01

**Authors:** 


Dr. James W. Casey, of Rochester, N. Y., died at his home November 18, 1907, aged 73 years. He graduated at the University of Buffalo in 1862, and served as assistant surgeon of the 105th volunteer infantry during the civil war. He was a member of several medical societies and served on the staff of St. Mary's Hospital for nearly forty years.

